# CD248 promotes migration and metastasis of osteosarcoma through ITGB1-mediated FAK-paxillin pathway activation

**DOI:** 10.1186/s12885-023-10731-7

**Published:** 2023-03-30

**Authors:** Shiqi Lu, Tong Lu, Jiayu Zhang, Lunbiao Gan, Xinjie Wu, Donghui Han, Keying Zhang, Chao Xu, Shaojie Liu, Weijun Qin, Fa Yang, Weihong Wen

**Affiliations:** 1 Xi’an Key Laboratory of Stem Cell and Regenerative Medicine, Institute of Medical Research, Northwestern Polytechnical University, 710072 Xi’an, Shaanxi China; 2 Department of Urology, Xijing Hospital, Air Force Medical University, Xi’an, China

**Keywords:** Endosialin/CD248/TEM-1, Osteosarcoma, Migration, Metastasis, ITGB1

## Abstract

**Background:**

Osteosarcoma (OS) is the most common malignant bone tumor with a high incidence in children and adolescents. Frequent tumor metastasis and high postoperative recurrence are the most common challenges in OS. However, detailed mechanism is largely unknown.

**Methods:**

We examined the expression of CD248 in OS tissue microarrays by immunohistochemistry (IHC) staining. We studied the biological function of CD248 in cell proliferation, invasion and migration of OS cells by CCK8 assay, transwell and wound healing assay. We also studied its function in the metastasis of OS in vivo. At last, we explored the potential mechanism how CD248 promotes OS metastasis by using RNA-seq, western blot, immunofluorescence staining and co-immunoprecipitation using CD248 knockdown OS cells.

**Results:**

CD248 was highly expressed in OS tissues and its high expression was correlated with pulmonary metastasis of OS. Knockdown of CD248 in OS cells significantly inhibited cell migration, invasion and metastasis, while had no obvious effect on cell proliferation. Lung metastasis in nude mice was significantly inhibited when CD248 was knocked down. Mechanistically, we found that CD248 could promote the interaction between ITGB1 and extracellular matrix (ECM) proteins like CYR61 and FN, which activated the FAK-paxillin pathway to promote the formation of focal adhesion and metastasis of OS.

**Conclusion:**

Our data showed that high CD248 expression is correlated with the metastatic potential of OS. CD248 may promote migration and metastasis through enhancing the interaction between ITGB1 and certain ECM proteins. Therefore, CD248 is a potential marker for diagnosis and effective target for the treatment of metastatic OS.

**Supplementary Information:**

The online version contains supplementary material available at 10.1186/s12885-023-10731-7.

## Introduction

Osteosarcoma (OS) is the most common malignant bone tumor with a high incidence in children and adolescents [1, 2]. At the initial diagnosis, about 10–15% of OS patients were found to have lung metastasis, and up to 80% of patients were found to have lung micro-metastasis [3, 4]. Currently, surgical removal and neoadjuvant chemotherapy are the mainstays of OS treatment. However, relapse and metastasis happen rapidly after treatment, and the 5-year survival rate was only 15-20% [5–7]. Therefore, it is with vital importance to explore the mechanisms and identify effective diagnostic markers and therapeutic targets for metastatic OS.

CD248, also known as endosialin or tumor endothelial marker 1 (TEM1), is a type I transmembrane glycoprotein [8]. Besides its specific expression in tumor stroma (such as CAFs and pericytes) of various tumors, CD248 has been found to be highly expressed in most kinds of sarcomas, including OS [9–12]. Furthermore, CD248 expression was significantly higher in all high-grade and metastatic sarcomas than low-grade sarcomas, thus CD248 was correlated with sarcoma metastasis [13]. Overexpression of CD248 in non-migrative OS cell line MG63 significantly promoted cell migration [14], and CD248-targeting antibody MORAb-004 could effectively inhibit the migration of OS cells, while didn’t affect cell proliferation [15]. These evidences indicate that CD248 may regulate the metastasis of OS, however, detailed mechanism is not yet known.

Extracellular matrix (ECM) can help tumor cells to form pseudopodia to facilitate cell metastasis [16]. Integrins are important components that mediate the communications between tumor cells and ECM [17]. When binding with ECM proteins, several integrins, such as integrin beta 1 (ITGB1), could activate multiple signaling pathways like focal adhesion kinase (FAK) and Src, to recruit various proteins to form focal adhesions and promote cell migration [18–20]. ITGB1 has been found to be the receptor of fibronectin (FN), collagen, laminin, etc. and has been shown to regulate the invasion and metastasis in several cancer types [21–25]. Interestingly, CD248 could also bind with FN, collagen type I (Col I), IV (Col IV) and MMRN2 to promote the migration of cancer cells [26, 27]. In addition, OS cells secrete Matrix metalloproteinases (MMPs) which are a large family of calcium-dependent zinc-containing endopeptidases, being responsible for tissue remodeling and degradation of extracellular matrix (ECM) [28]. Since both CD248 and ITGB1 can interact with ECM proteins and may regulate cell metastasis, whether they could work together to synergistically promote the metastasis of OS deserves further investigation.

In this study, we found that CD248 was highly expressed in OS tissues and its high expression was associated with pulmonary metastasis. Knockdown of CD248 resulted in attenuated OS cell migration and invasion both in vitro and in vivo, while CD248 overexpression accelerated cell migration and invasion. Mechanistically, RNA-seq results indicated that CD248 was involved in the regulation of focal adhesion and migration signaling pathways in OS cells. By using immunofluorescence (IF) staining, Western blot and co-immunoprecipitation (co-IP) experiments, we demonstrated that CD248 could enhance the interaction between ITGB1 and ECM proteins such as FN and CYR61, activate FAK-paxillin pathway, promote the formation of focal adhesion, thus promote OS metastasis. Therefore, we demonstrated that CD248 could promote cell migration and metastasis in OS, which might be used as effective diagnostic marker and therapeutic target for metastatic OS.

## Materials and methods

### IHC staining

Human OS and bone microarray, which contains 27 OS tissues and 6 adjacent normal bone tissues was obtained from Shanghai Zhuoli Biotechnology Company Ltd. Tissue microarray was dewaxed and rehydrated with xylene and graded ethanol. After process of antigen retrieval and the blockade of endogenous peroxidase, slide was then incubated with rabbit monoclonal CD248 antibody (#ab204914, Abcam) at 4℃ for overnight followed by the incubation with HRP-labeled secondary antibody for 30 min at room temperature. Slide was counterstained with hematoxylin before they were mounted with coverslips. IHC staining score of CD248 was determined according to the percentage (0, 0%; 1, < 10%; 2, 10–40%; 3, 40–70%;4, 70–100%) and intensity (0, no staining; 1, weak staining; 2, moderate staining, 3, strong staining) of positive area. For statistical analysis, samples with staining scores between 0 and 3 were grouped as “CD248-low” and samples with staining scores between 4 and 12 were grouped as “CD248-high”.

### Cell culture and transfection

Human OS cell lines SJSA-1, HOS, MG63, 143B, U2OS, Saos2, neuroblastoma cell line SK-N-AS, and Ewing’s sarcoma cell line A-673 were obtained from Shanghai cell bank, and were identified by Specialized Technology Resources (STR) to confirm that cells are free of mycoplasma chlamydia contamination. Cells were maintained in DMEM medium (Gibco, Thermo Fisher Scientific, USA) supplemented with 10% FBS (Gibco, Thermo Fisher Scientific, USA) and 1% penicillin-streptomycin (Gibco, Thermo Fisher Scientific, USA) at 37 °C with 5% CO_2_. CD248 specific siRNAs (referred to as siCD248 #1, siCD248 #2) and corresponding negative control (referred to as siCtrl) were synthesized by Zimmer. Plasmid pCMV3-SP-N-Myc-CD248 (referred to as OE-CD248) and corresponding negative control (called Control) were purchased from Yiqiao Shenzhou. OS cells were transfected with these siRNAs or plasmid using Lipofectamine 3000 (Invitrogen, USA) according to the manufacturer’s instructions. Target sequences of the siRNAs used in this study were: siCD248 #1, 5’- CCAAAUAUCCGGAGCUCUUTT-3’; siCD248 #2, 5’-GCGCAUCACUGACUGCUUTT-3’.

### ECM protein treatment

SJSA-1 cells were seeded into 6-well plates and transfected with siRNA, and cultured until cell density reached 90%. Culture medium were then removed and cells were washed with PBS before FN (2 µg/mL) or Poly-L-Lysine (0.1 mg/mL) solution was added and incubate at 37℃. FN or Poly-L-Lysine was then removed at indicated timepoints and cells were washed with PBS before cell lysis buffer was added to prepare cell lysates for Western blot. Untreated cells were used as 0 min control.

### Western blot

Cells were lysed in RIPA buffer supplemented with PMSF (Sparklade, China), separated by 8% SDS-PAGE, and transferred onto PVDF membranes (Millipore, Danvers, MA). After blocking with skim milk for 1 h, the membrane was incubated with the following primary antibodies at 4 °C for overnight: anti-CD248 (#ab204914, Abcam), anti-CYR61 (#14,479, CST), anti-paxillin (#ab32084, Abcam), anti-p-paxillin (#69,363, CST), anti-GAPDH (#10494-1 -AP, Proteintech), anti-AKT (#4691, CST), anti-p-AKT (#4060, CST), anti-FAK (#71,433, CST), anti-p-FAK (#8556, CST). Membranes were then incubated with HRP-conjugated secondary antibody (1:10,000; Sparklade) for 1 h at room temperature, followed by visualization by chemiluminescence.

### Real time-quantitative PCR (RT-qPCR)

Cells were harvested and total RNA was isolated using an RNA isolation kit (#R6834010000J28U100, Omegabio). Reverse transcription (RT) was performed using PrimeScript™ RT Master Mix (TaKaRa, Japan). QPCR was then performed using SYRB Green II kit (#DRR041A; TaKaRa, Japan). The following primers were used: human-CD248 forward (F): 5’-CTCCACACATTCGTGTTCGC-3’ and reverse (R): 5’-CTGCTACGCTCTCTTCCCAC-3’; and human-GAPDH-F: 5’-GCAACTAGGATGGTGTGGCT-3 ' and R: 5’-TCCCATTCCCCAGCTCTCATA-3’.

### Cell counting kit 8 (CCK8) assay

CCK8 assay kit (Mishushengwu, China) was used to measure cell proliferation. Approximately 3 × 10^3^ cells/well of transfected cells were seeded in 96-well plates and analysis was performed at indicated timepoint. Before detection, 10 µL CCK8 reagent was added to each well, and incubated at 37 ℃ for 1 h. Then, the absorbance at 450 nm was measured by a microplate reader.

### Transwell assay

Transwell chambers (Corning, USA) containing Matrigel gel (BD Biosciences, USA) were used to assess cell invasion, and chambers without Matrigel gel were used for migration assay. Cells that were transfected for 48 h were used to prepare cell suspension using serum-free medium, 200 µL of cell suspension (1 × 10^5^ cells) was added to the upper chamber, and 500 µL of DMEM medium containing 10% FBS was added to the lower chamber. After 48 h of incubation, invaded or migrated cells were fixed with 4% paraformaldehyde for 30 min and stained with 0.1% crystal violet for 15 min. The number of invaded and migrated cells were counted under the microscope.

### Wound healing assay

Cells that were transfected for 48 h were seeded on six-well plate at the density of 5 × 10^5^ cells per well. Twelve hours later, a scratch was made using a 200 µL pipette tip, then culture medium was changed into medium with 2% FBS. At this time, 0 h images were captured, plates were then returned to the incubator, and images were taken again after 24 and 48 h under a phase contrast microscope (Olympus, Japan).

### Immunofluorescence (IF) staining

When cells grown in confocal dishes reached 100% confluence, a scratch was made with a pipette tip, after 12 h cells were rinsed with PBS for 3 times, then fixed with 4% paraformaldehyde for 15 min. After being washed with PBS, cells were permeabilize with PBS containing 0.5% Triton X-100 for 20 min at room temperature. After being washed with PBS, cells were blocked in normal goat serum for 30 min at room temperature. Then, cells were incubated with diluted primary antibody at 4 °C for overnight. After washing with TBST, cells were incubated with diluted fluorescent antibodies for 1 h at room temperature. Cells were then incubated with DAPI for 5 min in the dark, washed with PBS and fixed with blocking solution containing anti-fluorescence quencher. Images were taken under a fluorescence microscope.

### Animal study

Animal experiments in this study were approved by the Laboratory Animal Welfare and Ethics Committee of Northwestern Polytechnical University. Sixteen BALB/c nude mice (male, 6 weeks old) were purchased from Unilever (Beijing, China) and maintained in a 12 h light/12 h dark cycle with free access to food and water. For subcutaneous inoculation, 1 × 10^6^ luciferase expressing CD248 knockdown or control SJSA-1 cells were subcutaneously injected into the flank of each mouse, and when palpable tumors appeared, bioluminescence imaging (BLI) was performed and tumor volume was measured every 3 days with a vernier caliper [V = (width^2^ × length / 2)]. For metastasis study, 1 × 10^6^ lentivirus-transfected CD248 knockdown or control SJSA-1 cells were injected into each mouse through tail vein and BLI was observed under the Xenogen IVIS Kinetic imaging system on the 8th day after injection. They were euthanized by intraperitoneal injection of sodium pentobarbital (50 mg/kg), the tumor tissues were collected and weight was measured.

### Cell adhesion assay

The 96-well plate was coated with five different ECM proteins (FN, 3 µg/cm^2^; Poly-L-Lysine, 0.05 mg/cm^2^; Collagen I, 10 µg/cm^2^; Vitronectin, 0.5 µg/cm^2^; Laminin, 5 µg/cm^2^) for 12 h. Then CD248 knockdown or control SJSA-1 cells was seeded on the coated 96-well plate and incubated at 37℃ for 2 h before cells were gently rinsed with PBS for 3 times. The quantity of adhered cells was detected using CCK8 assay kit, and cell adhesion rate was calculated using cultured same number of SJSA-1 cells as control.

### Co-immunoprecipitation (co-IP)

Cells were lysed at 4℃ in an immunoprecipitation lysis buffer containing protease inhibitor cocktail (Sparkjade, China). Cell lysates were incubated with corresponding antibody for 16 h at 4℃ followed by a 2 h incubation with Protein A/G beads (Santa Cruz Biotechnology, US) at 4℃. After three washes with IP lysis buffer, protein samples were collected by boiling in 1 × SDS loading buffer and subjected to standard SDS-PAGE and western blot.

### Statistical analysis

Statistical analysis was performed using GraphPad Prism 8. Quantitative data were presented as mean ± standard deviation (SD) or mean ± standard error of mean (SEM). Comparisons of the groups were analyzed by Student’s t-test or one-way ANOVA. P-values less than 0.05 were considered to be statistically significant.

## Results

### CD248 was highly expressed in OS tissues and was correlated with metastasis

**Fig. 1:**
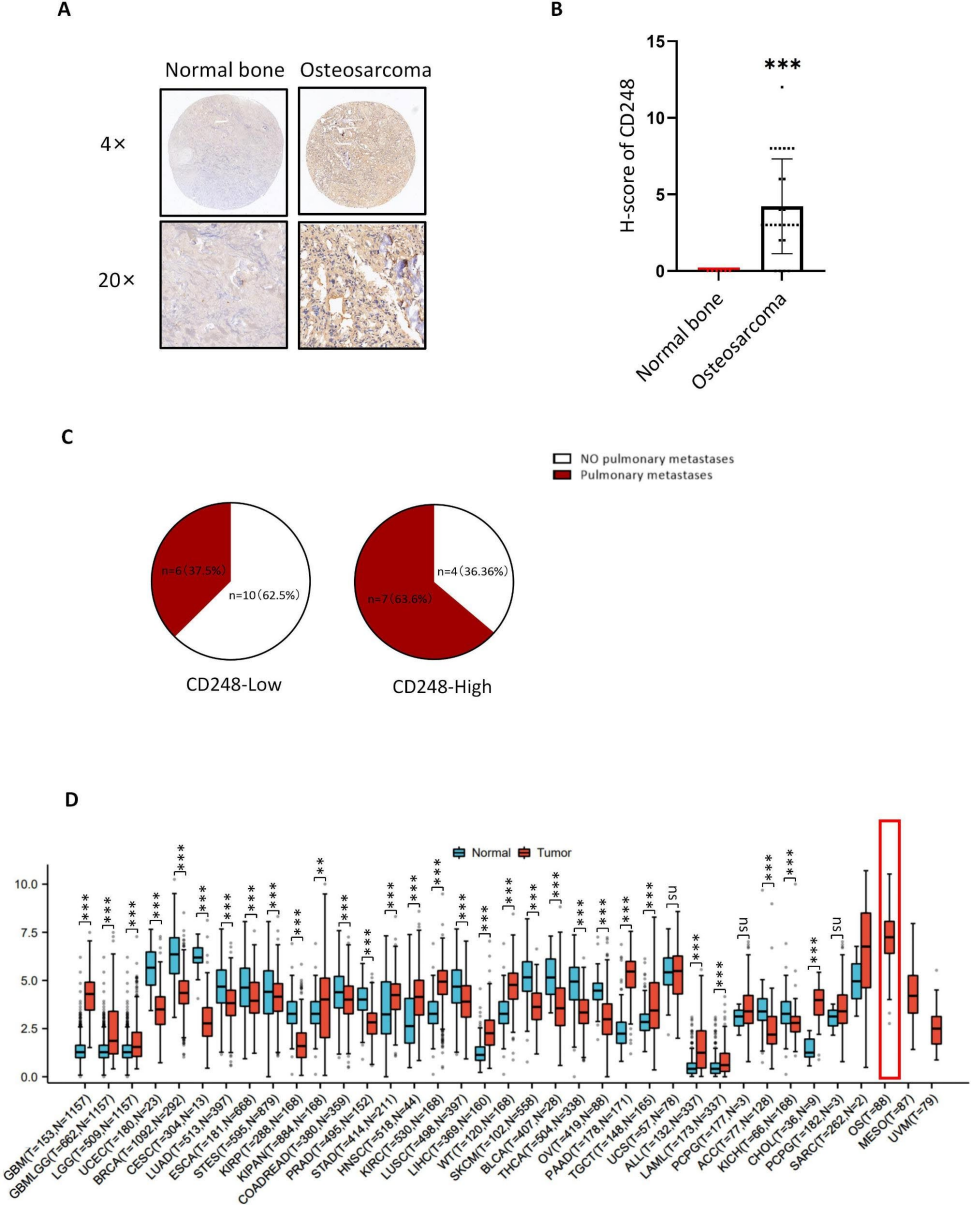
CD248 was highly expressed in OS tissues and was correlated with metastasis. **(A)** Representative IHC staining data to shown the expression of CD248 in OS tissues and normal bone tissues. **(B)** Histogram to show the IHC scores of CD248 in OS tissues and normal bone tissues. Data represent the mean ± SD. **(C)** Pie charts to show the proportions of pulmonary metastases in CD248-high and CD248-low OS patients. **(D)** Expression of CD248 in various tumor tissues and adjacent normal tissues in TCGA database. *P < 0.05, **P < 0.01, ***P < 0.0001

To examine the expression of CD248 in OS tissues, we performed IHC staining on OS tissue microarray which contains 27 OS tissues and 6 adjacent normal bone tissues. Results showed that CD248 was highly expressed in vast majority of OS tissues, but all the normal bone tissues didn’t show CD248 expression (Fig. 1A and B). Based on IHC staining score, we divided the OS tissues into two groups, that is CD248-high and CD248-low groups, and analyzed the correlation between CD248 expression and pulmonary metastasis. Results showed that 63.6% (7/11) of the CD248-high group had pulmonary metastasis, while 37.5% (6/16) of the CD248-low group had metastasis, indicating that high CD248 expression was correlated with OS metastasis (Table S1, Fig. 1C). We also analyzed expression of CD248 in different cancer types in TCGA, TARGET and GTEx databases [29, 30], which showed that OS had obviously high CD248 expression (Fig. 1D). These results demonstrate that CD248 is highly expressed in OS tissues and is correlated with metastasis.

### CD248 didn’t influence the proliferation of OS cells both in vitro and in vivo

**Fig. 2:**
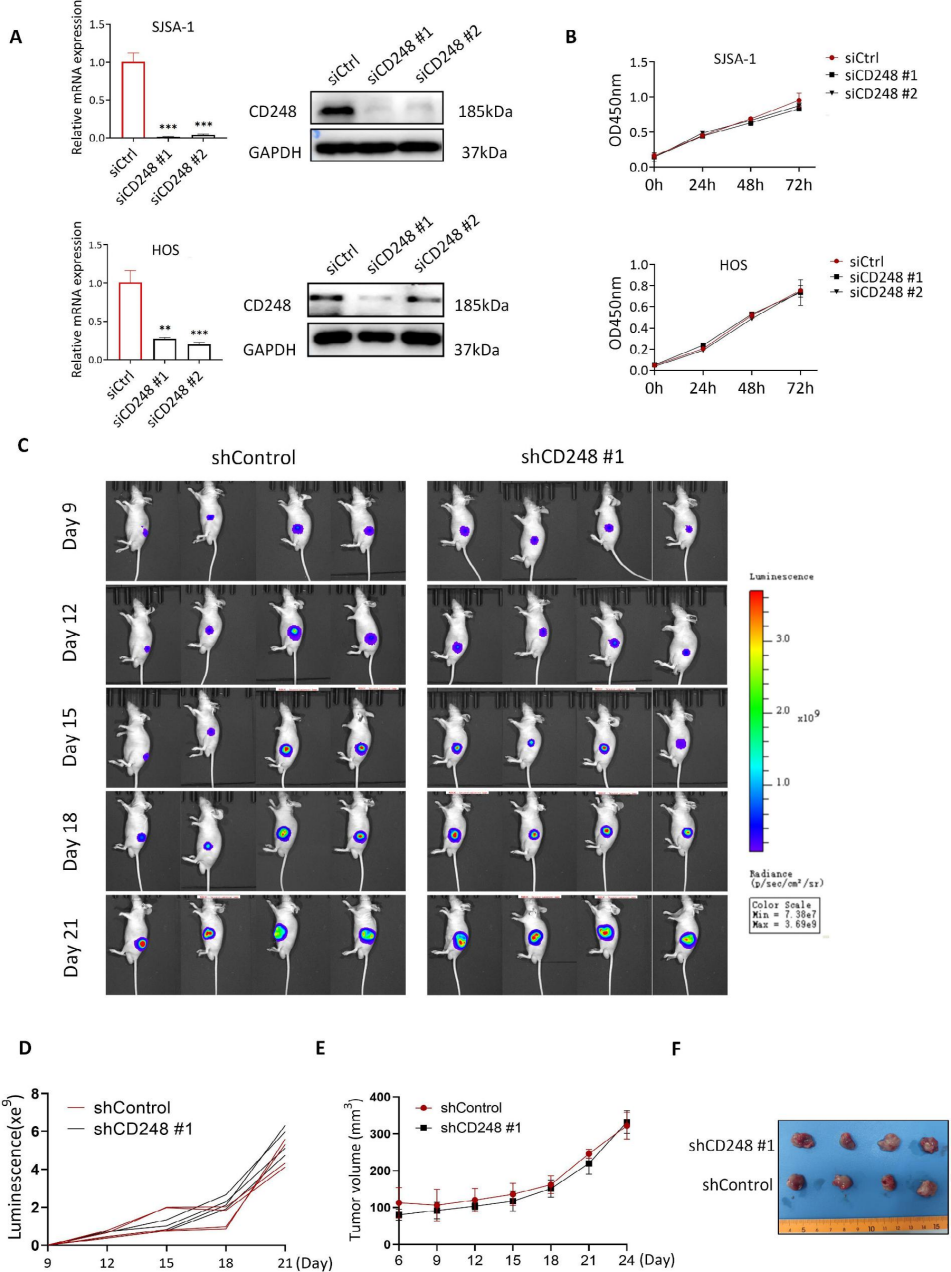
CD248 didn’t influence the proliferation of OS cells both in vitro and in vivo. **(A)** RT-qPCR and Western blot to show the knockdown efficiency of CD248 siRNA in SJSA-1 and HOS cells. **(B)** CCK8 assay to show the cell proliferation of CD248 knockdown and control SJSA-1 and HOS cells. **(C)** Bioluminescence imaging (BLI) to show the tumor growth of CD248 knockdown and control SJSA-1-luc cells. **(D)** BLI to show luminescent signals of the tumors in each mouse. **(E)** Growth curves of the CD248 knockdown and control SJSA-1 xenografts (n = 4) **(F)** Pictures of the CD248 knockdown and control SJSA-1 xenografts. The original blots/gels are presented in the Supplementary Material 2.

To study the biological function of CD248 in OS cells, we first examined the expression level of CD248 in eight different sarcoma cell lines, and found that SJSA-1, HOS, A-673, SK-N-AS cells had relatively high CD248 expression, while CD248 expression was relatively low in 143B, MG63, Saos2 and U2OS cells (Fig. S1A-S1B). Then we knocked down CD248 in SJSA-1 and HOS cells, which had high CD248 expression, and confirmed the knockdown efficiency by RT-qPCR and Western blot (Fig. 2A). CCK8 assay was performed to examine cell proliferation and results showed that knockdown of CD248 didn’t influence the proliferation of SJSA-1 and HOS cells (Fig. 2B). Then we confirmed the function of CD248 in OS cell proliferation in vivo by using stable CD248 knockdown SJSA-1-luc cells, which were infected by shRNA-expressing lentivirus. Knockdown efficiency of the shRNA-expressing lentivirus was also confirmed by RT-qPCR and Western blot (Fig. S2A-S2B). In vivo data showed that there was no difference in the tumor growth of subcutaneously inoculated CD248 knockdown or control SJSA-1-luc xenografts, as shown by the similar fluorescence signals examined by BLI, tumor growth curve and tumor volume (Fig. 2C-F). These findings indicate that CD248 does not influence the proliferation of OS cells in vitro and OS tumor growth in vivo.

### Knockdown of CD248 inhibited OS cell migration and invasion in vitro, and tumor metastasis in vivo

**Fig. 3:**
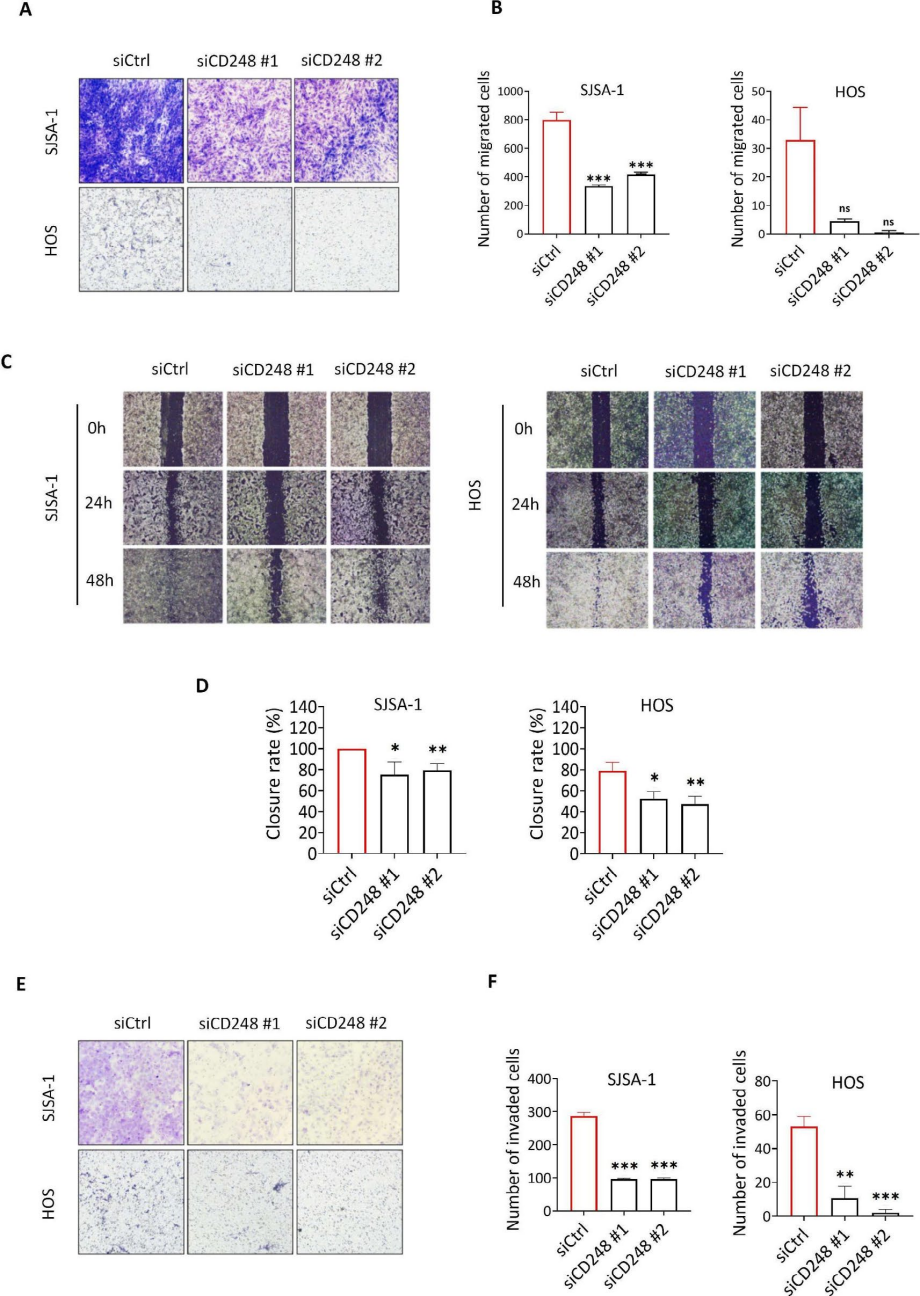
Knockdown of CD248 inhibited migration and invasion of OS cells in vitro. **(A)** Transwell assay to show the migration of CD248 knockdown and control SJSA-1 and HOS cells. **(B)** Statistical analysis of the number of migrated cells in A. **(C)** Transwell assay to show the invasion of CD248 knockdown and control SJSA-1 and HOS cells. **(D)** Statistical analysis of the number of invaded cells in C. **(E)** Wound healing assay to show the migration of CD248 knockdown or control SJSA-1 and HOS cells. **(F)** Statistical analysis to show the closure rate of CD248 knockdown or control SJSA-1 and HOS cells in E. Representative images were shown. *P < 0.05, **P < 0.01, ***P < 0.0001

**Fig. 4:**
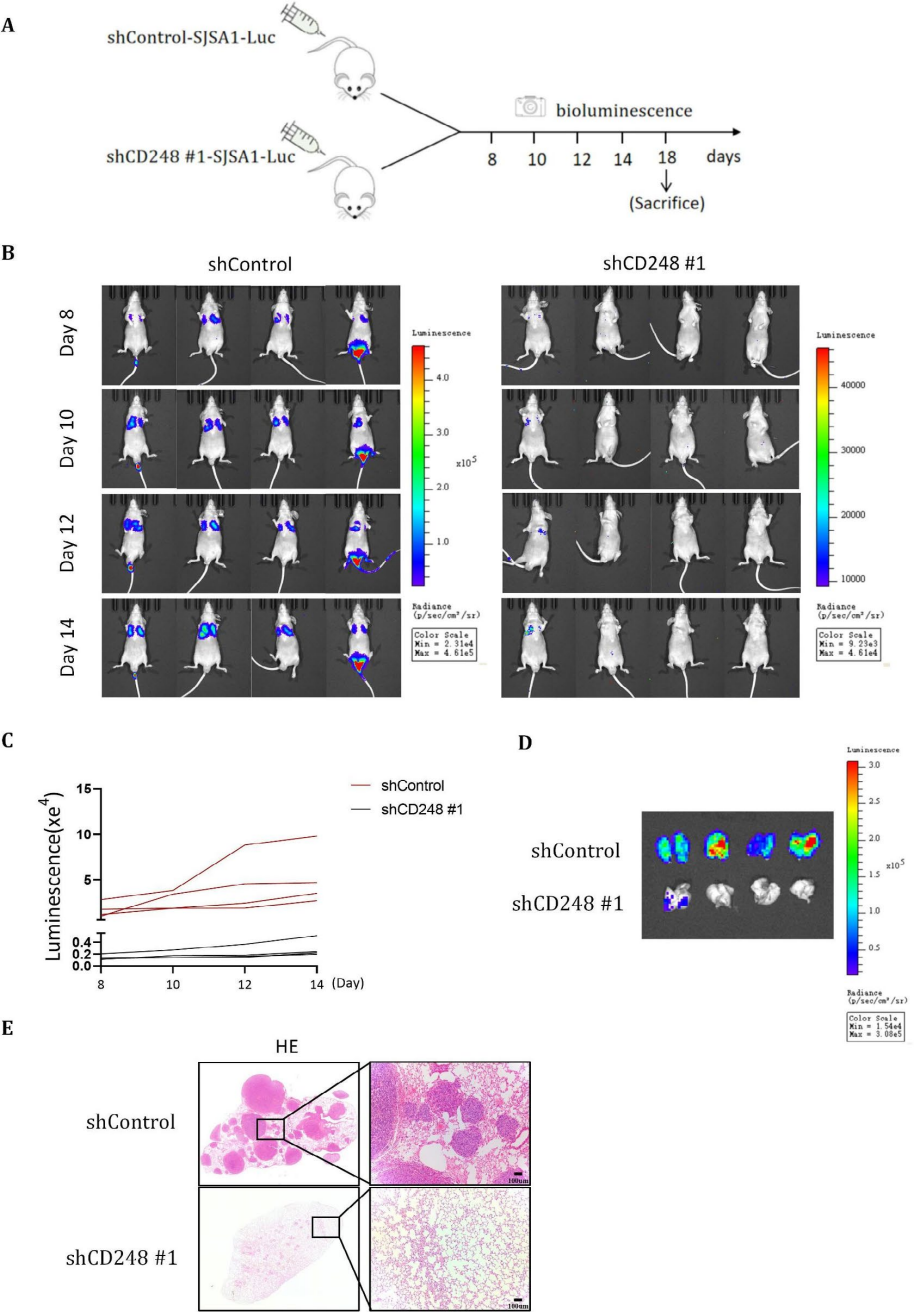
Knockdown of CD248 inhibited the metastasis of OS cells in vivo. **(A)** Schematic diagram of experiment to examine the metastasis of OS cells in vivo. **(B)** BLI to show the metastasis of tail vein injected CD248 knockdown and control SJSA-1-luc cells. **(C)** BLI data of each mouse at different time points after tail vein injection. **(D)** BLI to show the luminescent signals in the lung tissues of each mouse. **(E)** HE staining of the lung tissues to show the metastatic nodules. Representative images were shown

Next, we examined whether CD248 may regulate the migration and invasion of OS cells. As shown by wound healing and transwell results, both CD248 knockdown SJSA-1 and HOS cells showed significant decreased migratory capacity, compared with control group (Fig. 3A-D). Similarly, as shown by the results of transwell with Matrigel, the invasive ability of CD248 knockdown SJSA-1 and HOS cells was also inhibited (Fig. 3E-F). We also confirmed the function of CD248 in cell migration using CD248-overexpressing MG63 cells, which originally has very low CD248 expression. Results of transwell showed that overexpression of CD248 significantly enhanced the migration of MG63 cells (Fig. S3A-S3C). By using the tail vein injection metastasis model, we further evaluated the effect of CD248 on the metastasis of SJSA-1 cells in vivo (Fig. 4A). BLI results showed that the control group had obvious lung metastasis on the eighth day after tail vein injection, however, the CD248 knockdown group almost did not show any metastasis signal (Fig. 4B-C). At the end of the experiment, mice were sacrificed and main organs were dissected and fluorescence signal was observed. Results showed that only the lung tissues of the control group had fluorescence signal, no obvious metastasis was found in all the organs in CD248 knockdown group (Fig. 4D, Fig. S4). We also examined the metastatic nodules in lung tissue by HE staining and found that the lung tissue of the control group had multiple metastatic nodules, while no metastatic nodule was observed in CD248 knockdown group (Fig. 4E). These findings confirm that CD248 can significantly promote the migration and invasion of OS cells in vitro and OS tumor metastasis in vivo.

### CD248 was involved in multiple cell adhesion and migration pathways

**Fig. 5:**
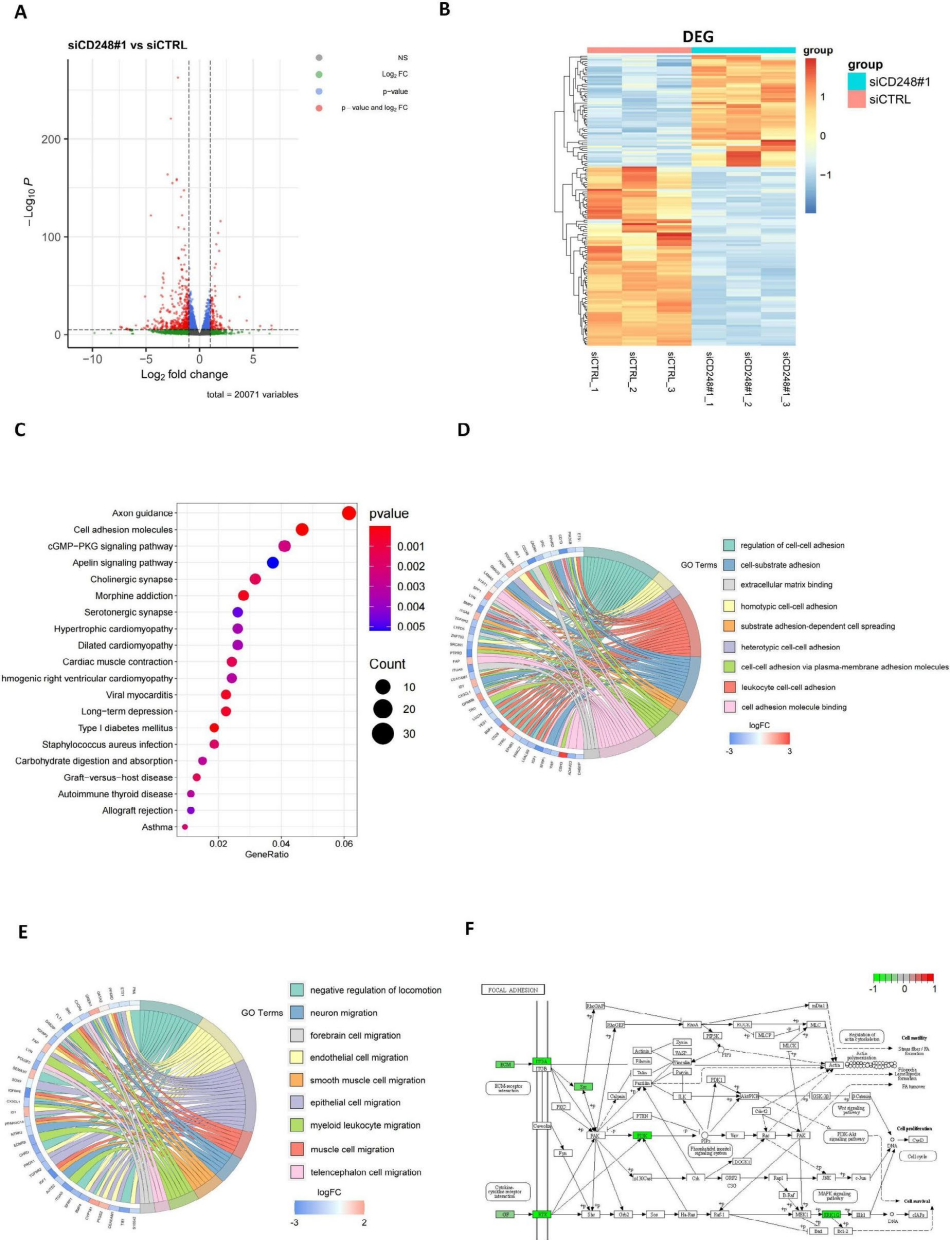
CD248 was involved in multiple cell adhesion and migration pathways. **(A)** Volcano map to show the differentially expressed genes in CD248 knockdown and control SJSA-1 cells. **(B)** Hierarchical clustering analysis of the differentially expressed genes. **(C)** KEGG pathway enrichment analysis of the differentially expressed genes. **(D, E)** Chord diagrams of GO enrichment analysis. The left semicircle represented names of the genes, and the right semicircle represents the functions that the genes may involve. **(F)** Focal adhesion pathway retrieved from the KEGG database [47–49]. Differentially expressed genes were labeled by color spectrum. GO, Gene Ontology; KEGG, Kyoto Encyclopedia of Genes and Genomes; DEGs, differentially expressed genes

To investigate the potential mechanism how CD248 promotes OS cell migration, invasion and metastasis, we performed RNA-seq using RNA from CD248 knockdown and control SJSA-1 cells. Results showed that knockdown of CD248 had great influence on gene expression profile, with 2713 genes down-regulated and 2000 genes up-regulated, using 2-fold as a cut-off value (Fig. 5A-B). We further analyzed the differentially expressed genes using KEGG pathway enrichment analysis and the top 20 enriched pathways were shown in Fig. 5C. Among them, cell adhesion molecules ranked second and was consistent with our findings. Since cell adhesion is an important step in tumor metastasis [31], therefore, we further analyzed the enrichment and expression of these differentially expressed genes in cell adhesion and migration pathways through GO analysis. Results showed that multiple cell adhesion-related pathways were enriched and most of these adhesion-related genes were also down-regulated in CD248 knockdown cells (Fig. 5D). We also analyzed cell migration pathway, and obtained similar results (Fig. 5E). During analysis, we noticed that Src kinase, which is a key regulator in focal adhesion and migration, was significantly down-regulated in CD248 knockdown cells (Fig. 5E). Therefore, we focused on the changes of molecules in focal adhesion pathway and found that some key molecules (ITGA, Src, PI3K, ERK1/2) were also significantly down-regulated in CD248 knockdown cells (Fig. 5F). These results confirm that CD248 regulates multiple cell adhesion and migration pathways, of which the focal adhesion pathway is most significant.

### CD248 activated the ECM-stimulated FAK-paxillin pathway and enhance the formation of focal adhesion

**Fig. 6:**
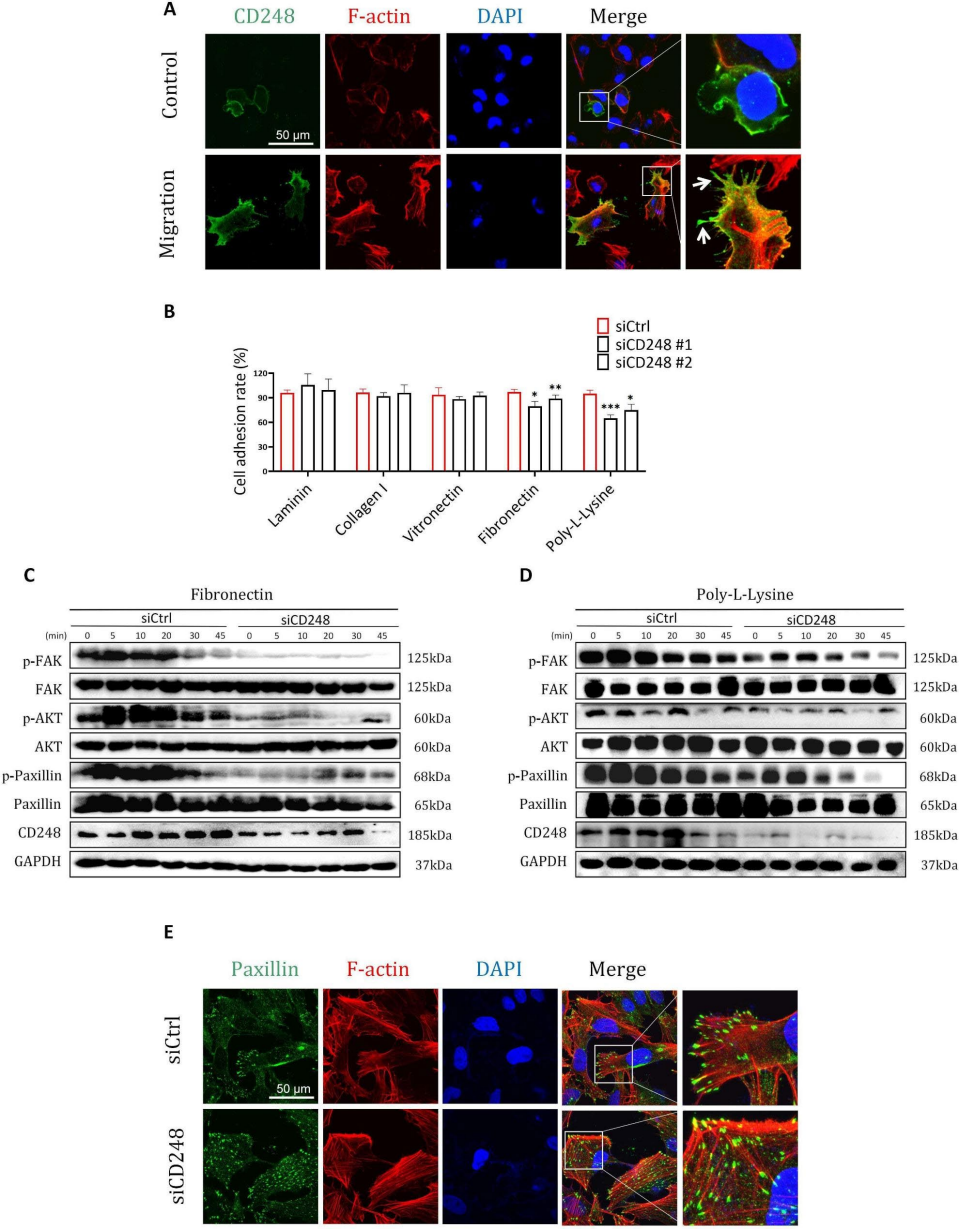
CD248 activated the ECM-stimulated FAK-paxillin pathway and enhance the formation of focal adhesion. **(A)** IF staining of CD248 and F-actin in SJSA-1 cells near or far away from the scratch. White arrows indicated the position of filiform pseudopodia. **(B)** Adhesion assay to show the adhesion of CD248 knockdown and control SJSA-1 cells with different ECM proteins. **(C)** Western blot analysis to show the activation of FAK-paxillin pathway in CD248 knockdown and control SJSA-1 cells after incubation with FN for different timepoints. Phosphorylation sites we examined were pFAK (Tyr397), pPaxillin (Tyr118) and pAKT (Ser473). **(D)** Western blot analysis to show the activation of FAK-paxillin pathway in CD248 knockdown and control SJSA-1 cells after incubation with Poly-L-Lysine for different timepoints. Phosphorylation sites: pFAK (Tyr397), pPaxillin (Tyr118), pAKT (Ser473). **(E)** IF staining of paxillin and F-actin in CD248 knockdown and control SJSA-1 cells after they were migrated on confocal dishes for 24 h. Scale bar = 50 μm. Representative images were shown. *P < 0.05, **P < 0.01, ***P < 0.0001. The original blots/gels are presented in the Supplementary Material 2.

Since CD248 is closely related with focal adhesion pathway, we examined the co-localization of CD248 and focal adhesion protein F-actin by IF staining during cell migration. Cells at the edge of the scratch were considered to be in an active migration state, and cells far away from the scratch were considered to be in a relatively static state. We found that in migrative cells, CD248 was aggregated in the bulge of the cells and colocalized with F-actin, which is a main component to form filopodia, while in relatively static cells CD248 was diffusely distributed on cell membrane (Fig. 6A). These results suggested that CD248 might be involved in the formation of focal adhesion during migration. We then examined whether knockdown of CD248 may influent cell adhesion to different ECM proteins, and found that cell adhesion to FN were significantly decreased after CD248 was knocked down, while cell adhesion to other ECM proteins like laminin, collagen I and vitronectin was not influenced, indicating that CD248 is involved in cell adhesion to specific ECM proteins. Poly-L-Lysine was used as positive control since its function in enhancing cell adhesion was well known (Fig. 6B). Then we examined the activation of the focal adhesion pathway after cells were treated with FN or Poly-L-Lysine. Results showed that after treatment with FN or Poly-L-Lysine, the phosphorylation of FAK, AKT and paxilin in CD248 knockdown cells was significantly inhibited compared with control cells (Fig. 6C-D). Furthermore, IF staining results showed that knockdown of CD248 also resulted in reduced number of paxillin puncta on cell membrane (Fig. 6E), indicating impaired focal adhesion formation and reduced cell migration. Thus, these results demonstrate that CD248 can activate ECM protein stimulated FAK-paxillin pathway to promote the formation of focal adhesion.

### CD248 enhanced the interaction between ITGB1 and ECM proteins

**Fig. 7:**
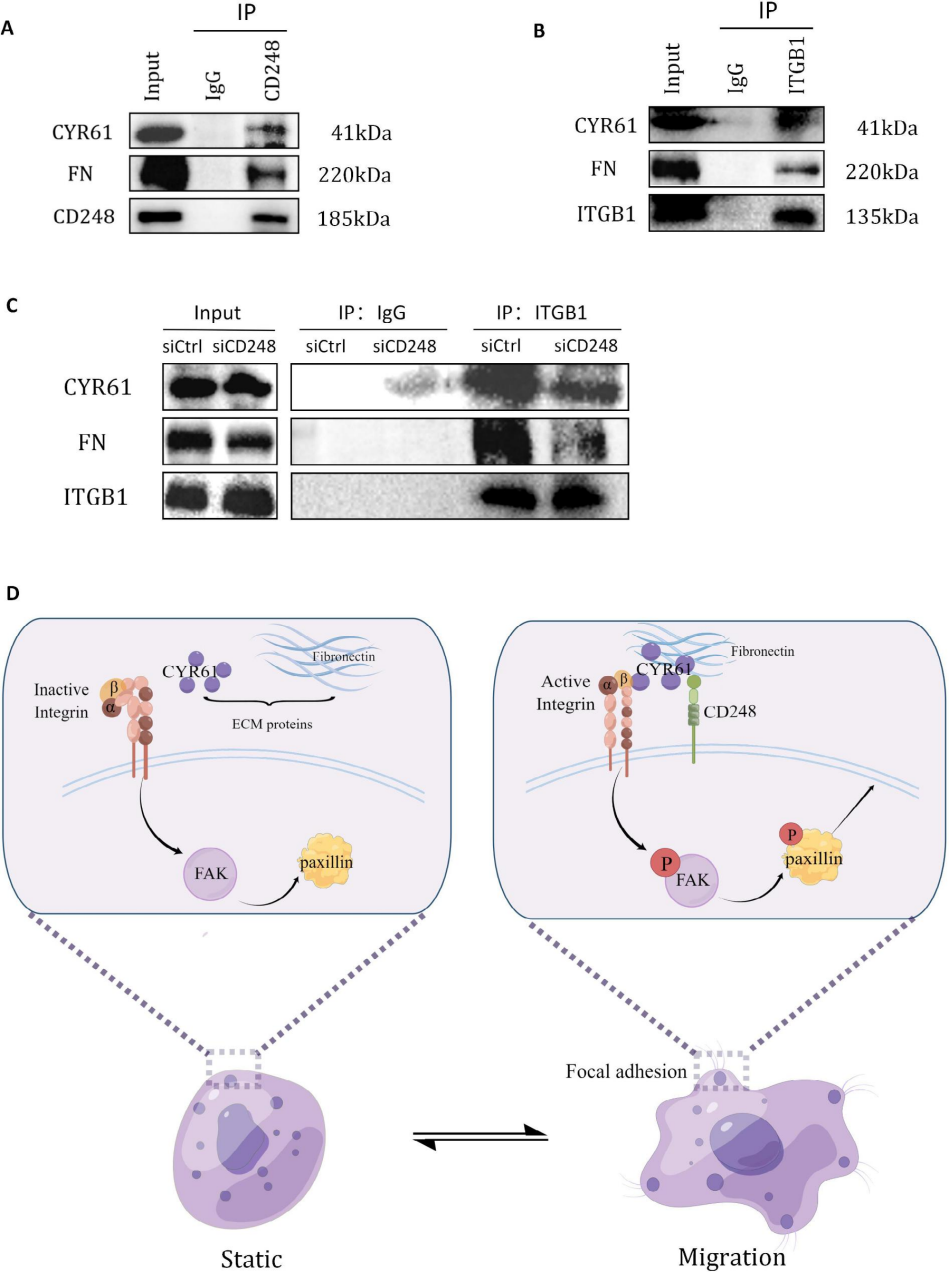
CD248 enhanced the interaction between ITGB1 and ECM proteins. **(A)** Co-IP experiments to show the interaction between endogenous CD248 and CYR61 or FN in SJSA-1 cells. **(B)** Co-IP experiments to show the interaction between endogenous ITGB1 and CYR61 or FN in SJSA-1 cells. **(C)** Co-IP experiments to show the interaction between endogenous ITGB1 and CYR61 or FN in CD248 knockdown and control SJSA-1 cells. **(D)** Schematic diagram to show the proposed function of CD248 in OS metastasis. The original blots/gels are presented in the Supplementary Material 2.

Since integrins are important components that mediate the communications between tumor cells and ECM proteins, and after stimulation by ECM proteins, ITGB1 can activate multiple signaling pathways such as FAK and Src to recruit various proteins to form focal adhesions and promote cell migration. Next, we explored whether CD248 may play a role in the interaction between ITGB1 and ECM proteins. Previous studies have shown that ECM proteins FN and CYR61 are the ligands of ITGB1 to promote the activation of FAK and cell migration, andCD248 has also been found to interact with FN and CYR61 [26, 32–34]. So, we first confirmed the interaction between CD248 and FN or CYR61 by co-IP experiments in SJSA-1 cells, and also the interaction between ITGB1 and FN or CYR61 (Fig. 7A and B). Then we examined the interaction between ITGB1 and FN or CYR61 in CD248 knockdown cells, and found that, indeed, the binding of ITGB1 to CYR61 or FN was obviously inhibited (Fig. 7C). These results demonstrate that CD248 can promote the interaction between ITGB1 and ECM proteins such as FN and CYR61. Thus, we proposed a working model how CD248 promotes OS cell migration, that is, during cell migration, increased CD248 expression promote the interaction between ITGB1 and ECM proteins, activate the FAK-paxillin pathway, promote the translocation of paxillin to form focal adhesion, thus promote cell migration and metastasis in OS (Fig. 7D).

## Discussion

Osteosarcoma (OS) is the most common bone malignancy, especially in children and adolescent. Approximately 10 to 15% of newly diagnosed OS patients have metastatic disease, primarily in the lung. The 5-year survival rate is approximately 60% in patients with localized OS, but is only 20% in patients with metastases or recurrent disease [6, 7]. Since most patients with recurrent disease have pulmonary metastases, surgical removal usually entails resection of pulmonary disease. However, current treatment, such as surgical resection followed by systemic chemotherapy could not increase overall survival, long-term survival rate is still below 20% [1]. Thus, novel mechanisms and effective biomarkers for metastasis of OS are urgently needed.

CD248 is a transmembrane glycoprotein that belongs to the C-type lectin-like receptor family. Plenty of studies have found that CD248 is highly expressed in most kinds of sarcomas, including OS [9, 11], and CD248 has been found to be related with cell migration and metastasis. Overexpression of CD248 in non-migrative OS cells MG63 cells could significantly enhance their migratory velocity, which could be further enhanced upon the addition of PDGF, a known chemoattractant [14]. Studies have also shown that metastatic OS had higher CD248 expression, and most metastatic OSs (7/8) had positive CD248 expression, while only a small percentage of non-metastatic OSs (1/10) had CD248 expression. The anti-CD248 antibody MORAb-004 could effectively inhibit the migration of OS cells, but didn’t influence their proliferation and differentiation [15]. In addition, intravenous injection of human OS cell SJSA-1, which has pretty high CD248 expression, resulted in lung metastasis [13]. This evidence indicates that CD248 could regulate the metastasis of OS, however, detailed mechanism is not known.

Some studies have shown that CD248 could regulate cell adhesion and metastasis in some other cancer types. For example, one study reported that CD248-positive pericytes can promote the infiltration of tumor cells into blood vessels and distant metastasis in a cell-contact-dependent manner [35]. Another study demonstrated that CD248 can directly bind with ECM proteins, such as fibronectin (FN) and collagen types I and IV, and overexpression of CD248 can promote cell adhesion to FN and enhance cell migration through Matrigel [26]. These studies suggest that CD248 may promote cell adhesion and metastasis through cell-cell or cell-matrix interactions.

Extracellular matrix (ECM) proteins play essential roles in tumor metastasis through regulating different intracellular signaling pathways [36]. Integrins are the major transducers of extracellular cues, which trigger downstream changes in gene expression to regulate cell adhesion, invasion and migration [37]. Several integrins have been found to be overexpressed in various cancer cell types and activate tumor cell invasion and metastasis upon binding with their ECM protein ligands [38]. Among different integrins, integrin beta 1 (ITGB1) has been shown to bind with certain ECM proteins like FN, collagen, laminin, et al., thus activate multiple signaling pathways such as FAK and Src to promote cell adhesion, invasion and metastasis in several cancer types [18–25, 39].

It has been shown that cancer cells use amoeboid migration as the preferred migratory strategy. And the amoeboid characteristics promote cancer cell metastasis, during which cancer cells may form protrusions at the leading edge of migrative direction, which is called pseudopodia or filopodia [40]. To ensure cancer cells to go directional migration after the sensing of directional cues, branched and linear actin filament will array in the leading edge and align with the direction of movement. And the contractile arrays of actin form stress fibers that are mechanically coupled to the substrate through integrin-based focal adhesions [41, 42]. Cytoskeletal forces are linked to the ECM through transmembrane integrin heterodimers that cluster to form focal adhesions [43]. Paxillin has been shown to play a central role during the formation of focal adhesions through recruiting a variety of proteins and signaling molecules [44]. And FAK-mediated paxillin phosphorylation is an important process for the formation of focal adhesions [45]. It is notable that OS contains abundant osteoid ECM, and that abnormal signaling and structural components of the ECM contribute to progression and metastasis of OS [46].

In this study, we first demonstrated that OSs with high CD248 expression are prone to have pulmonary metastasis. Then we confirmed that CD248 indeed could regulate OS cell migration and invasion both in vitro and in vivo, while didn’t influence the proliferation of OS cells. We also found that during OS cell migration, CD248 accumulated at filopodia, and regulated the location of paxillin and the formation of focal adhesion, indicating that CD248 plays an important role in the directional migration of OS cells. For mechanism study, by using RNA-seq and co-IP experiments, we demonstrated that CD248 could enhance the interaction between ITGB1 and ECM proteins such as FN and CYR61, thus activate FAK-paxillin pathway and promote cell migration and metastasis in OS.

In our study, although we provided a potential working model how CD248 promote OS cell migration, however, there still has limitations. First, in most cases, osteosarcoma develops intramedullary, however, in our animal study, OS cells were either inoculated in the flank of nude mice or injected through tail vain, therefore they could not exactly mimic the natural progress of OS in humans. Second, the number of clinical samples we examined was relatively low, larger number of samples are still needed to validate the correlation between CD248 expression and pulmonary metastasis. Third, according to our RNA-seq data, CD248 may regulate multiple signaling pathways that is related with tumor progression. At last, although we provided a potential mechanism which is dependent on ITGB1, however, CD248 may also promotes OS metastasis through ITGB1 independent mechanism since Poly-L-Lysine treatment, which could not induce integrin clustering, could also activate FAK-paxillin pathway. Thus, more studies are still needed to find out whether CD248 may promote OS metastasis through other mechanisms.

In conclusion, our study confirmed that endosialin was highly expressed in OS, and was correlated with pulmonary metastasis. CD248 could promote OS cell invasion, migration and metastasis, but had no obvious effect on cell proliferation. CD248 could enhance the interaction between ITGB1 and ECM proteins to activate FAK-paxillin pathway, promote the formation of focal adhesion, thus promote metastasis of OS cells. CD248 could be used as a diagnostic marker and effective target for the treatment for metastatic OS.

## Electronic supplementary material

Below is the link to the electronic supplementary material.

Supplementary Material 1

Supplementary Material 2

Supplementary Material 3

Supplementary Material 4

Supplementary Material 5

Supplementary Material 6

## Data Availability

The data supporting the finding of this study are available within the article and are available from the corresponding authors on reasonable request. The datasets presented in this study can be found in online repositories. The names of the repository/repositories and accession number(s) can be found below: https://www.ncbi.nlm.nih.gov/, PRJNA890330.
