# Supplementary material for: CD248 promotes migration and metastasis of osteosarcoma through ITGB1-mediated FAK-paxillin pathway activation
Source: BMC Cancer. 2023 Mar 30;23:290. doi: 10.1186/s12885-023-10731-7 (PMC10061858; doi:10.1186/s12885-023-10731-7)
Supplement: Supplementary file 1 — Supplementary Material 1 [file 12885_2023_10731_MOESM1_ESM.pdf]

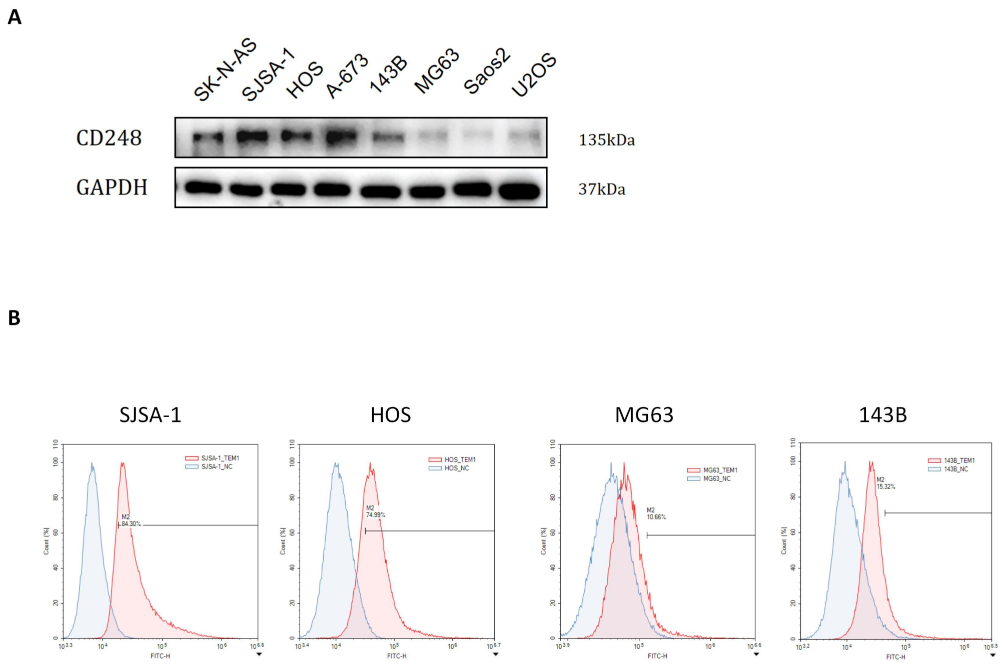

Supplementary figure 1. Expression of CD248 in sarcoma cell lines.
(A) Western blot to examine the expression of CD248 in different sarcoma cell lines.
(B) Flow cytometry to confirm the expression of CD248 in four OS cell lines.
