# Supplementary material for: CD248 promotes migration and metastasis of osteosarcoma through ITGB1-mediated FAK-paxillin pathway activation
Source: BMC Cancer. 2023 Mar 30;23:290. doi: 10.1186/s12885-023-10731-7 (PMC10061858; doi:10.1186/s12885-023-10731-7)
Supplement: Supplementary file 2 — Supplementary Material 2 [file 12885_2023_10731_MOESM2_ESM.pdf]

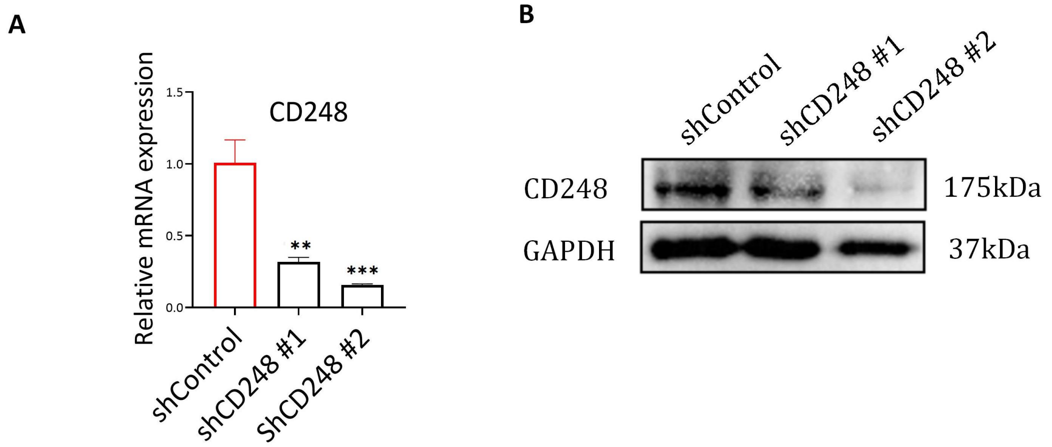

Supplementary figure 2. RT-qPCR and Western blot to confirm the knockdown efficiency of CD248 shRNA in SJSA-1 cells
