# Supplementary material for: CD248 promotes migration and metastasis of osteosarcoma through ITGB1-mediated FAK-paxillin pathway activation
Source: BMC Cancer. 2023 Mar 30;23:290. doi: 10.1186/s12885-023-10731-7 (PMC10061858; doi:10.1186/s12885-023-10731-7)
Supplement: Supplementary file 3 — Supplementary Material 3 [file 12885_2023_10731_MOESM3_ESM.pdf]

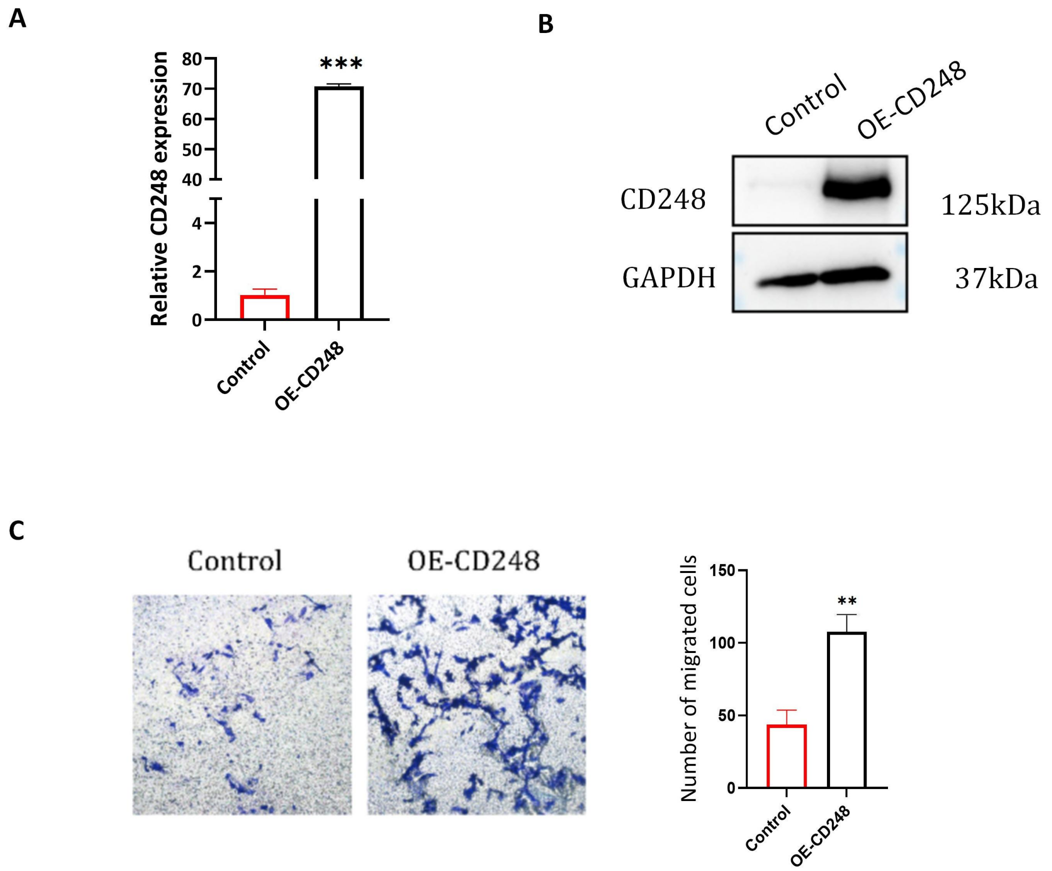

Supplementary figure 3. Overexpression of CD248 promotes invasion and metastasis of MG63 cells.

(A, B) RT-qPCR and Western blot to examine the expression of CD248 in CD248-overexpressing MG63 cells. (C) Left panel, transwell assay to show the migration of CD248-overexpressing MG63 cells. Right panel, statistical analysis of the number of migrated cells. Representative images were shown. **P <0.01.
