# Supplementary material for: CD248 promotes migration and metastasis of osteosarcoma through ITGB1-mediated FAK-paxillin pathway activation
Source: BMC Cancer. 2023 Mar 30;23:290. doi: 10.1186/s12885-023-10731-7 (PMC10061858; doi:10.1186/s12885-023-10731-7)
Supplement: Supplementary file 4 — Supplementary Material 4 [file 12885_2023_10731_MOESM4_ESM.pdf]

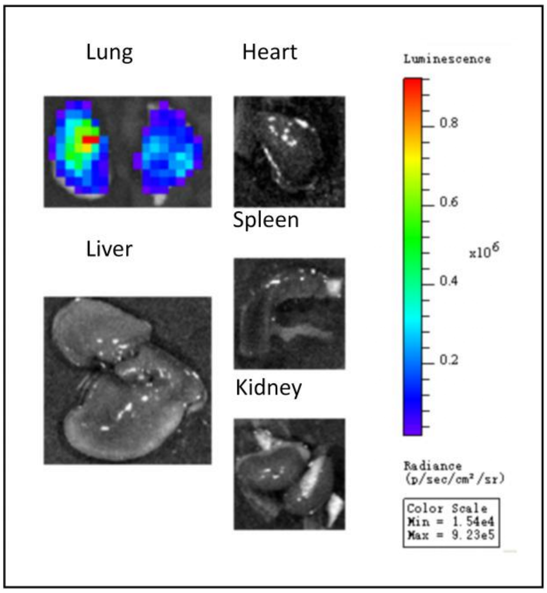

Supplementary figure 4. No obvious metastasis was found in other organs except lung after tail vein injection of SJSA-Luc cells.

BLI of different organs isolated from nude mice after tail vein injection of SJSA-Luc cells.
