# Supplementary material for: CD248 promotes migration and metastasis of osteosarcoma through ITGB1-mediated FAK-paxillin pathway activation
Source: BMC Cancer. 2023 Mar 30;23:290. doi: 10.1186/s12885-023-10731-7 (PMC10061858; doi:10.1186/s12885-023-10731-7)
Supplement: Supplementary file 5 — Supplementary Material 5 [file 12885_2023_10731_MOESM5_ESM.pdf]

Number	Gender	Age	Clinical diagnosis	Pathological diagnosis	IHC staining score	Metastasis
1	Male	25	Recurrent osteosarcoma of right fibula	Osteosarcoma	8	No
2	Male	22	Right proximal tibia Osteosarcoma	Osteosarcoma	3	No
3	Female	60	Osteosarcoma	Osteosarcoma	0	No
4	Male	15	Right lower femur osteosarcoma	Sclerosing osteosarcoma	0	No
5	Female	21	Left distal femur osteosarcoma	Chondromyxoid osteosarcoma	8	Pulmonary metastasis
6	Male	12	Right fibular head osteosarcoma	Osteosarcoma	3	No
7	Female	18	Left distal femur osteosarcoma	Osteosarcoma	3	No
8	Male	12	Right mid-femur osteosarcoma	Osteosarcoma	3	No
9	Female	18	Right mid-femur osteosarcoma	Osteosarcoma	3	Pulmonary metastasis
10	Female	12	Right mid-femur osteosarcoma	Osteosarcoma	8	No
11	Female	23	Postoperative recurrence of left humeral osteosarcoma	Recurrent osteosarcoma	12	Pulmonary metastasis
12	Male	18	Left lower femur bone tumor	Osteosarcoma	2	No
13	Male	18	Left humerus osteosarcoma	Osteosarcoma	0	Pulmonary metastasis
14	Female	15	Right distal femur osteosarcoma	Osteosarcoma	3	Pulmonary metastasis
15	Female	49	Right distal femur osteosarcoma	Osteosarcoma	4	No
16	Male	13	Right distal femur osteosarcoma	Osteosarcoma	0	Pulmonary metastasis
17	Male	55	Left distal femur osteosarcoma	Osteosarcoma	6	Pulmonary metastasis
18	Male	13	Left femoral osteosarcoma with arterial bone cyst	Osteosarcoma	8	Pulmonary metastasis
19	Male	51	Intramedullary well-differentiated osteosarcoma of the left femur	Intramedullary well-differentiated osteosarcoma	6	Pulmonary metastasis
20	Male	12	Right tibia osteosarcoma	Osteosarcoma	3	Pulmonary metastasis
21	Male	19	Right proximal tibia osteosarcoma	Osteosarcoma	8	Pulmonary metastasis
22	Female	43	Recurrence of left distal femur osteosarcoma	Well-differentiated osteosarcoma	4	Pulmonary metastasis
23	Male	20	Right distal femur osteosarcoma	Osteosarcoma	3	No
24	Female	14	Left proximal humerus osteosarcoma	Common osteosarcoma	2	No
25	Male	20	Right proximal humerus osteosarcoma	Osteosarcoma	3	No
26	Male	67	Right iliac osteosarcoma	Osteosarcoma	3	Pulmonary metastasis
27	Female	14	Left proximal humerus osteosarcoma	Osteosarcoma	8	No

Tables1. Patient information of Human OS and bone microarray
