# Supplementary figures and images for: CD248 promotes migration and metastasis of osteosarcoma through ITGB1-mediated FAK-paxillin pathway activation
Source: BMC Cancer. 2023 Mar 30;23:290. doi: 10.1186/s12885-023-10731-7 (PMC10061858; doi:10.1186/s12885-023-10731-7)

Figure2 A

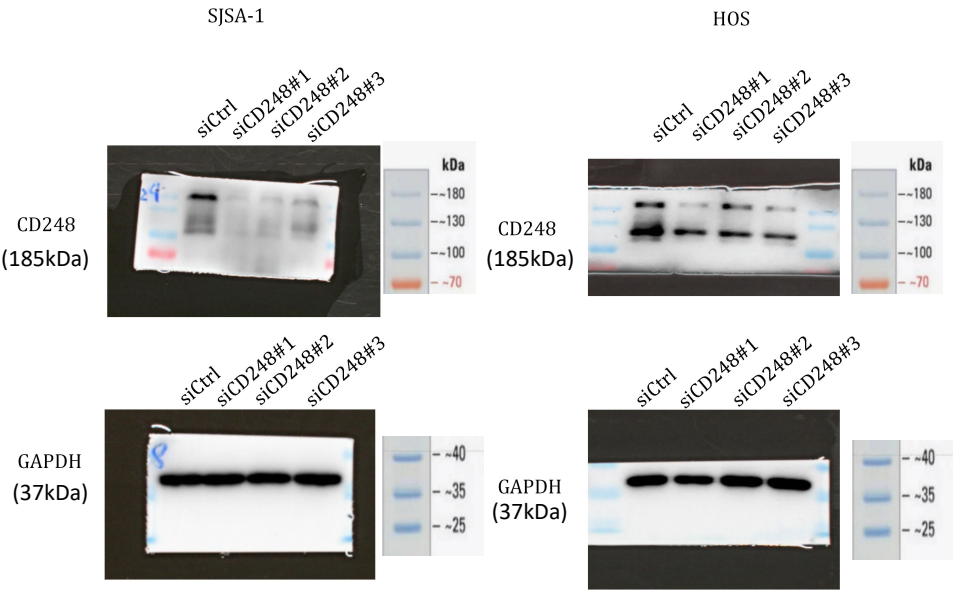

Figure6 C

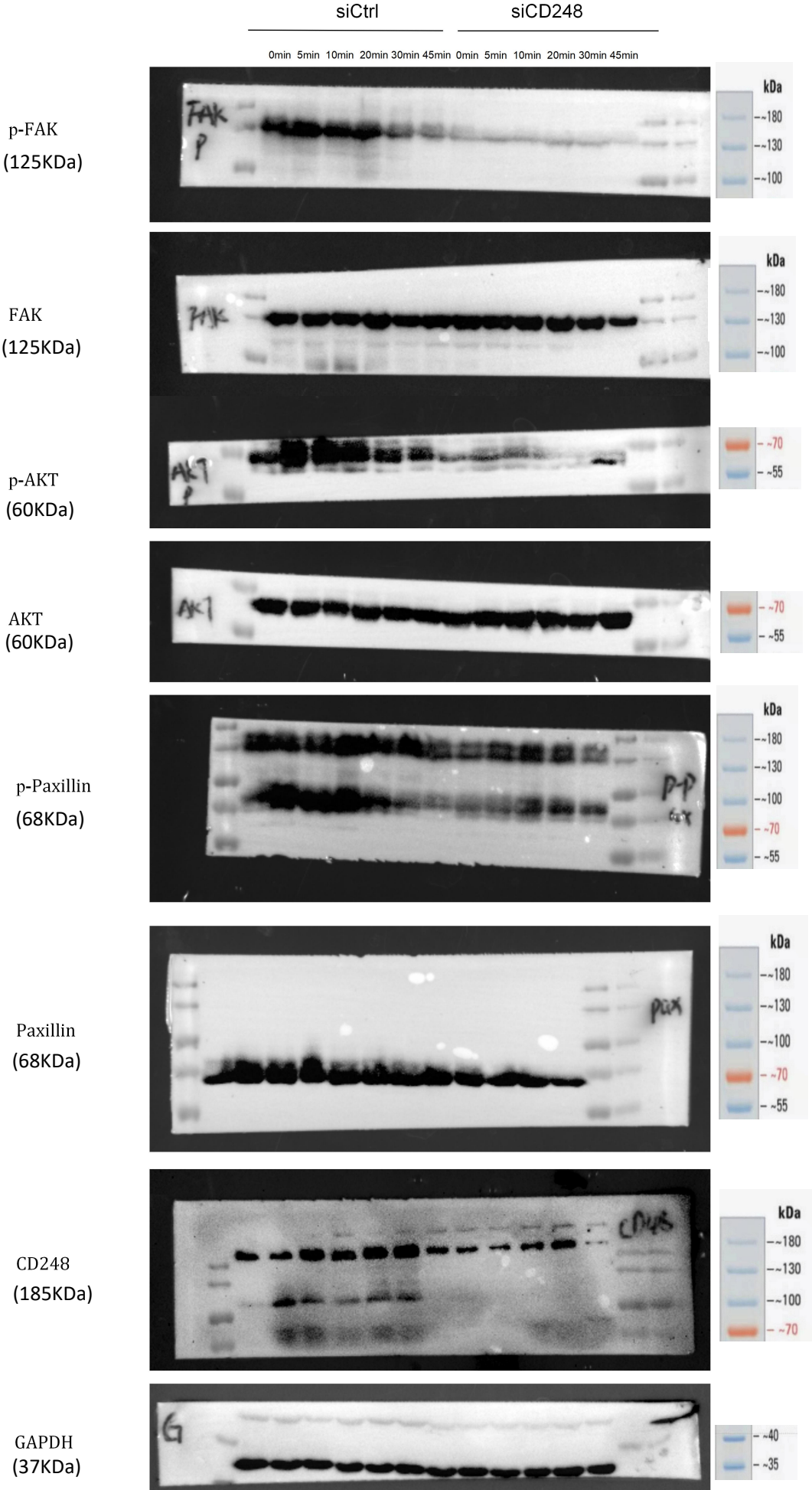

Figure6 D

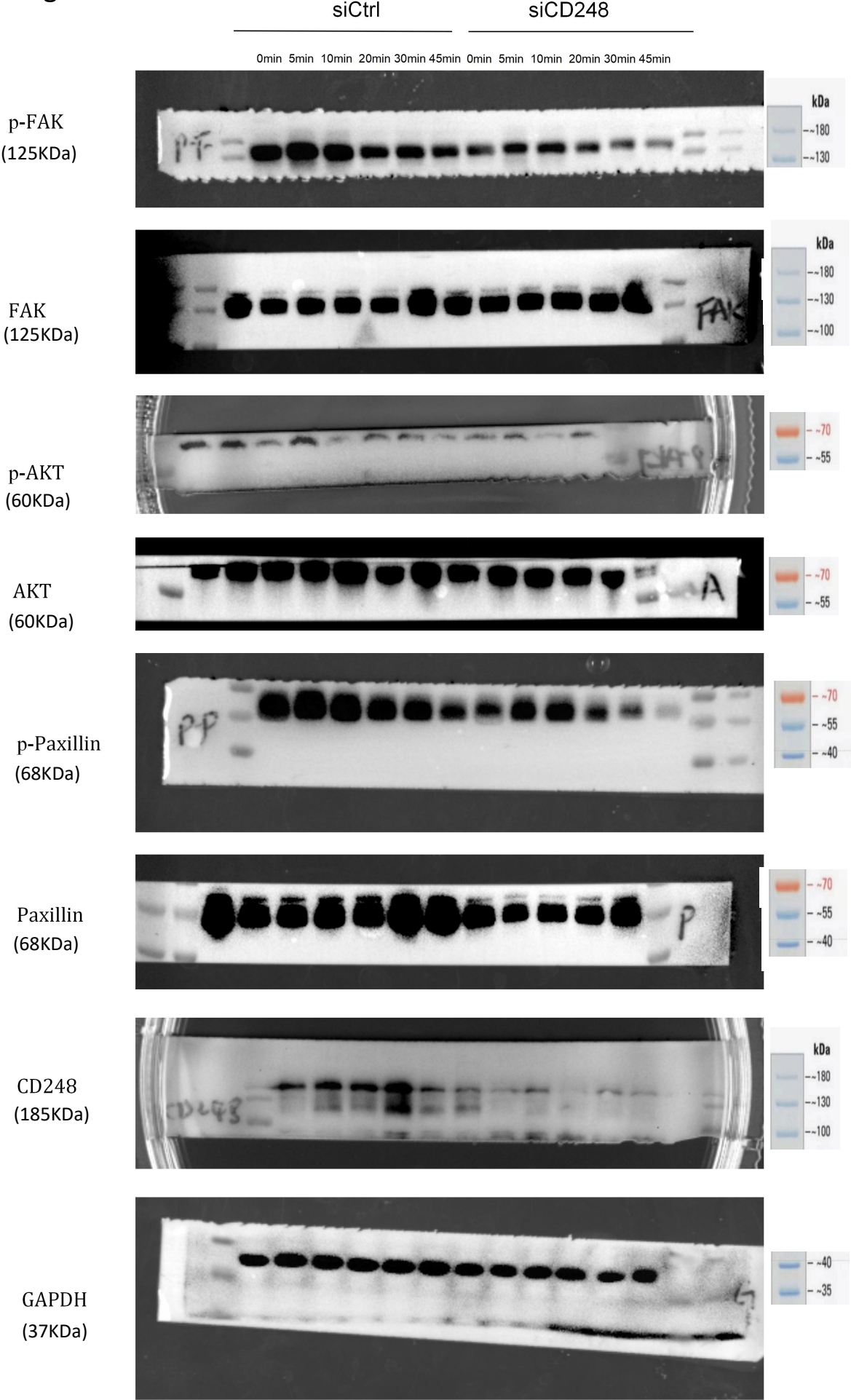

Figure7 A

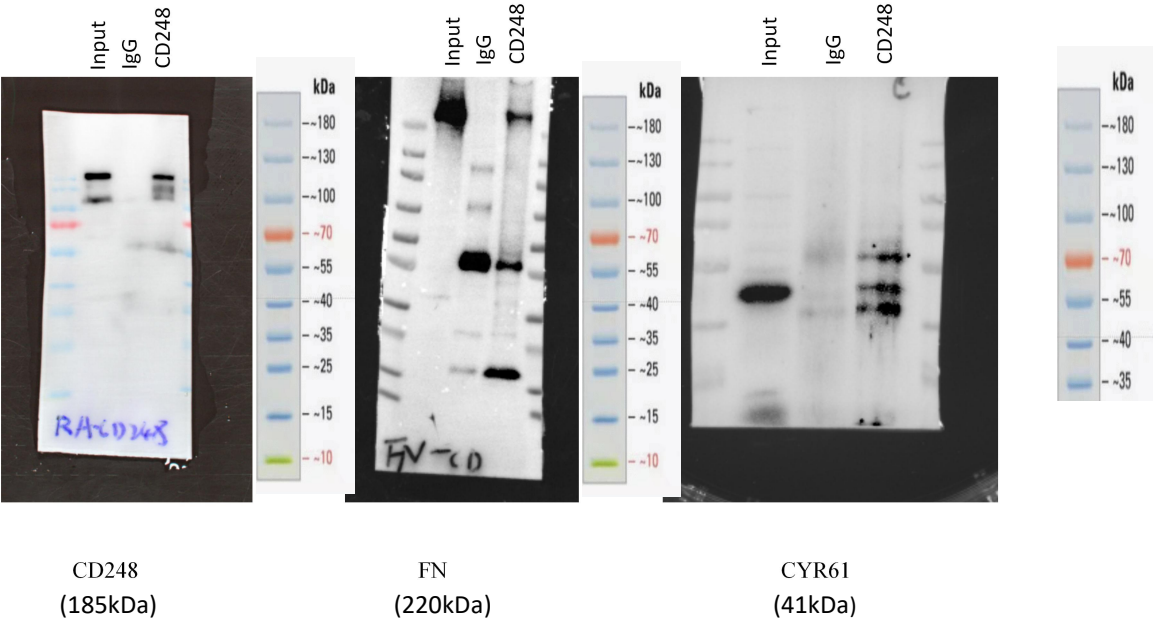

Figure7 B

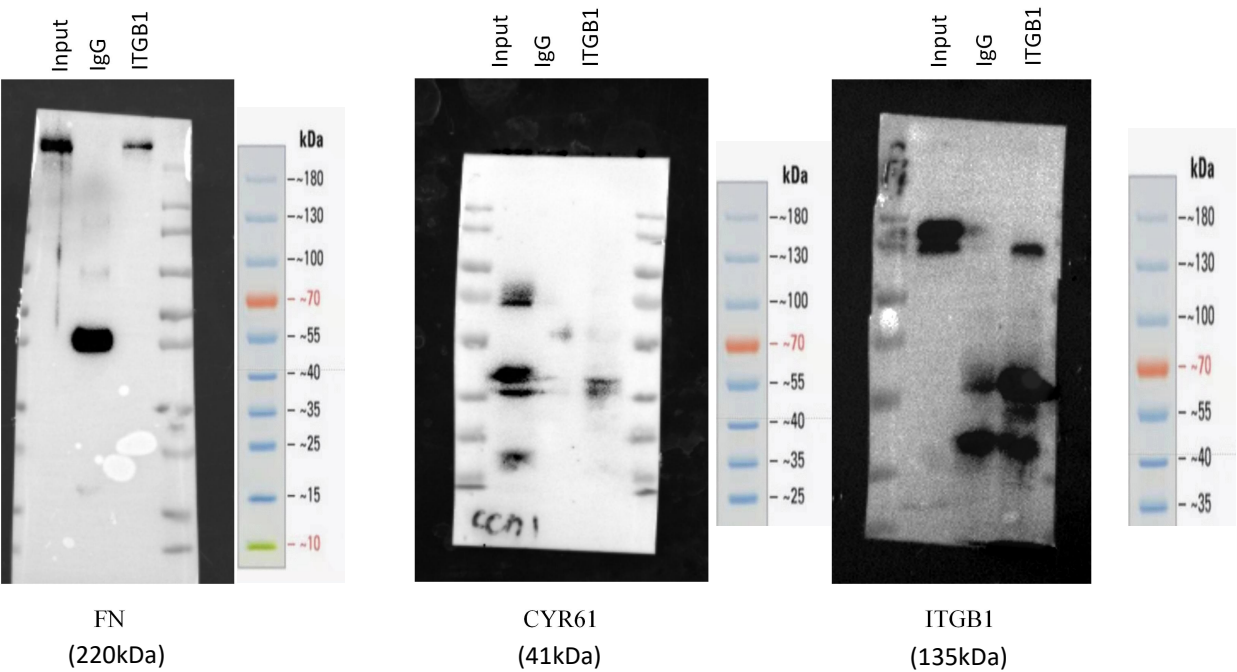

Figure7 C

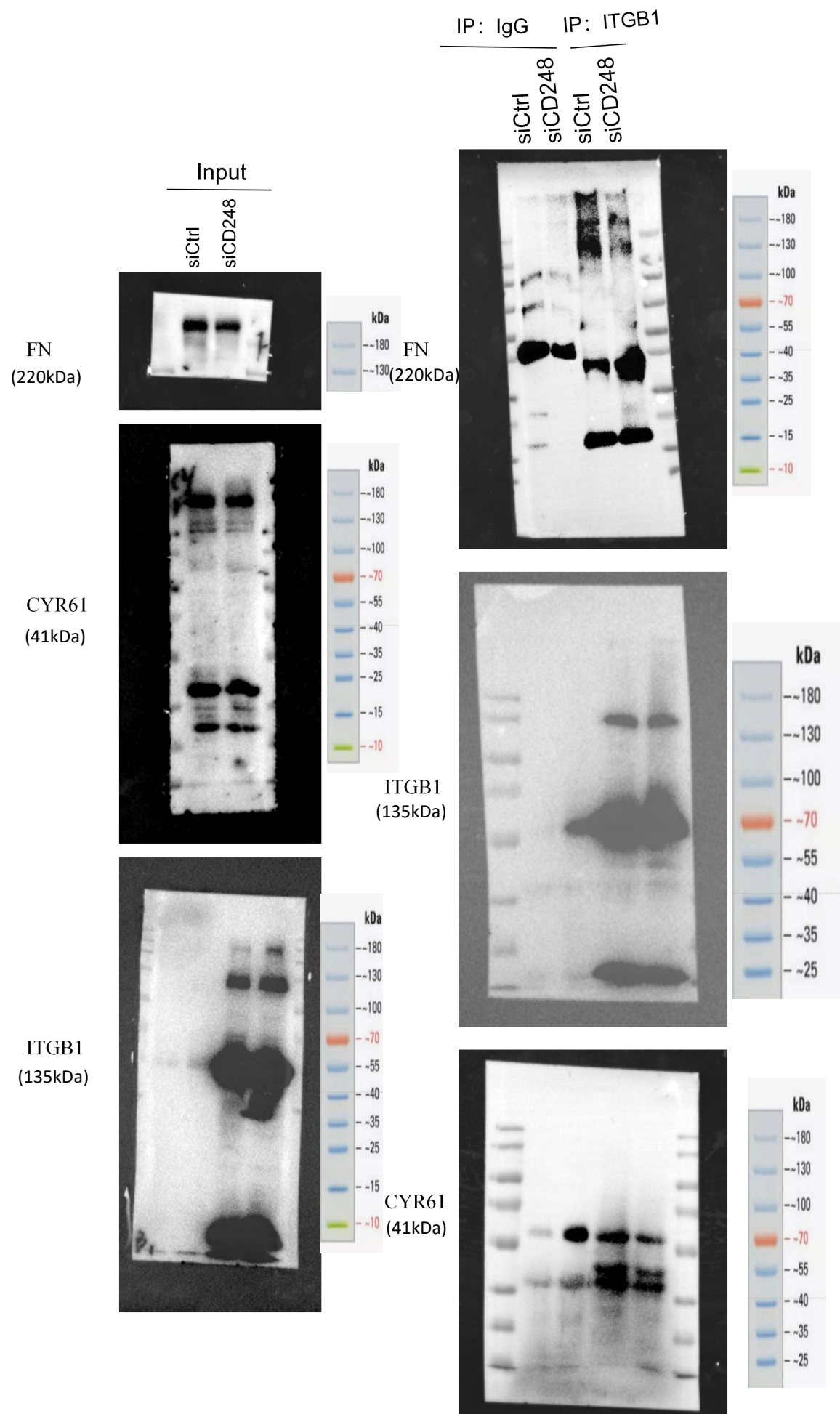

FigureS1 A

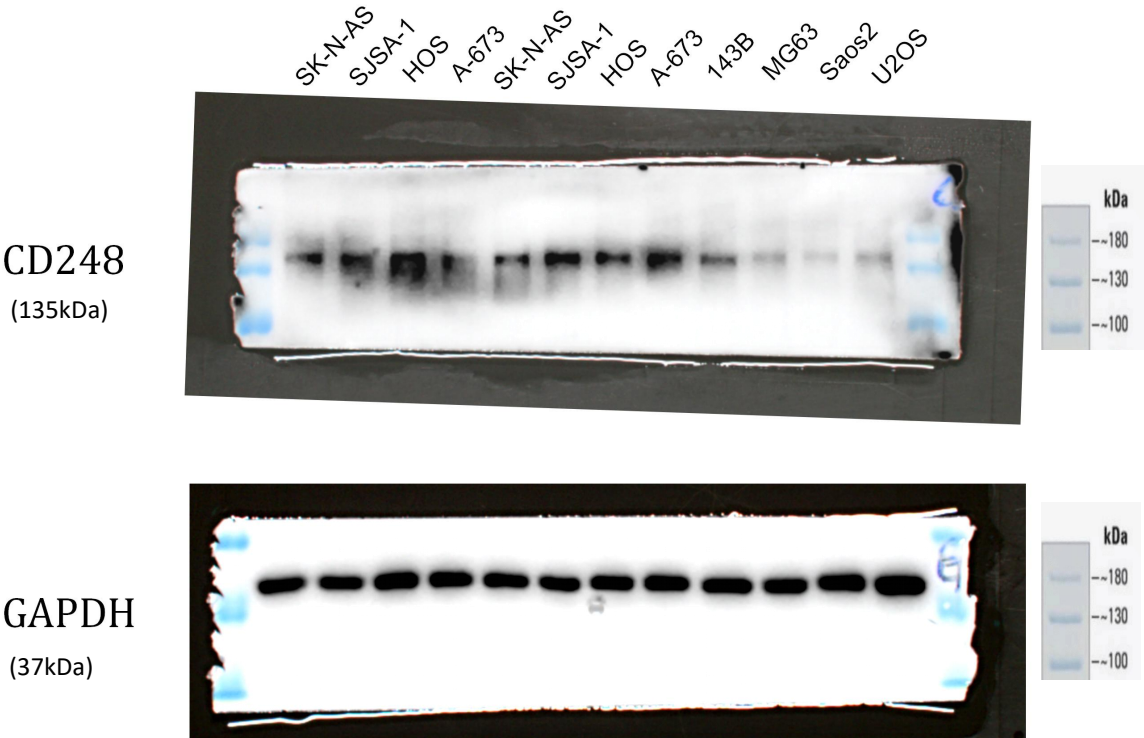

FigureS2 B

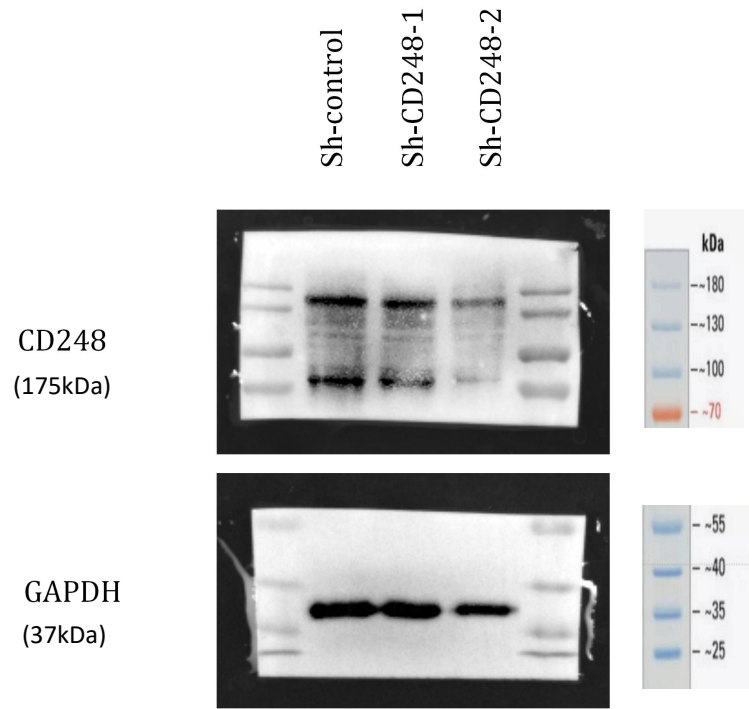

FigureS3 B

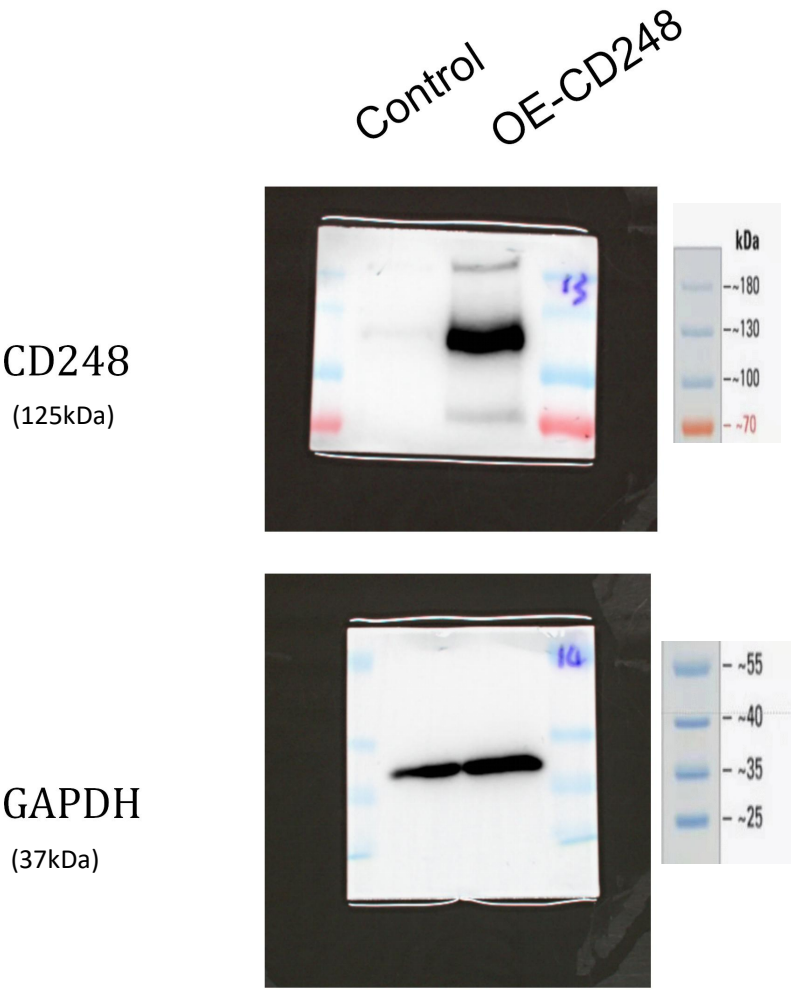

Supplement: Supplementary file 6 — Supplementary Material 6 [file 12885_2023_10731_MOESM6_ESM.pdf]
